# Measurement of portal vein indices and splenic index by ultrasound and their association with gastroesophageal varices in cirrhosis of liver

**DOI:** 10.1097/MS9.0000000000001483

**Published:** 2023-11-07

**Authors:** Shailendra Katwal, Mukhtar A. Ansari, Sundar Suwal, Surendra Rayamajhi, Prasoon Ghimire, Aastha Ghimire

**Affiliations:** a Department of Radiology, Dadeldhura Subregional Hospital, Dadeldhura; b Department of Radiology, National Medical college, Birgunj; c Department of Radiology, Maharajgunj Medical College; d Patan Academy of Health Science, Kathmandu; e Department of Radiology, Dhaulagiri Hospital, Baglung; f Department of Radiology, Lumbini Province Hospital, Butwol, Nepal

**Keywords:** congestion index, endoscopy, portal hypertension, spleen size, upper gastrointestinal bleeding

## Abstract

**Background and Objectives:**

Esophageal and gastric fundic varices are common in liver cirrhosis patients. Ultrasound with the Doppler study assesses liver cirrhosis severity, measuring portal vein and splenic indices’ association with gastroesophageal varices.

**Methodology:**

This study was conducted on 64 subjects with sonographic features of chronic liver disease who were referred for routine follow-up scans. Portal vein diameter, average velocity, splenic index, congestion index (CI), and portal vein area and velocity were measured.

**Result:**

Subjects with gastroesophageal varices had significantly larger portal vein diameters (14.7±1.64 mm) compared to those without varices (12.05±1.26 mm) (*P*<0.05). Conversely, subjects without varices exhibited a higher portal vein velocity of (17.9±0.6 cm/s) than with varices (13.91±2.01 cm/s) (*P*=0.0005). The splenic index was higher in subjects with varices (1120±494 cm^3^) than those without varices (419 cm^3^) (*P*<0.05). The CI was also higher in subjects with varices. Portal vein velocity showed the highest sensitivity (94%) with a cutoff of 19 cm/s, while the CI had the highest diagnostic accuracy (93.75%) with a cutoff of 0.10 cm xsec. The splenic index demonstrated a sensitivity of 92.85% and diagnostic accuracy of 92.18% with a cutoff of 480 cm^3^. The splenic index followed by the CI is found to be a better predictor of esophageal varices (area under the curve of 96.8 and 96%, respectively).

**Conclusion:**

Ultrasonographic assessment of the portal vein and spleen is a reliable, noninvasive method for predicting gastroesophageal varices in liver cirrhosis. The splenic index and CI have high diagnostic accuracy.

## Introduction

Highlights

- Ultrasound with Doppler can be utilized to measure portal vein and splenic indices in liver cirrhosis patients with or without gastroesophageal varices.
- Subjects with varices exhibited larger portal vein diameter, lower portal vein velocity, and a higher splenic index and congestion index.
- These ultrasound indices provide a reliable and accurate method for predicting gastroesophageal varices in liver cirrhosis.

Gastroesophageal varices are a leading cause of fatal upper gastrointestinal bleeding worldwide, especially in 50% of cirrhosis cases^1^. Gastric varices are the cause of 10–30% of all variceal bleeds, and up to 90% rebleed after spontaneous hemostasis^2^. Portal hypertension complicates cirrhosis, leading to ascites, splenomegaly, and gastroesophageal varices. Portal hypertension has prehepatic, intrahepatic, and posthepatic causes.

Ultrasound is a noninvasive, first-line tool for diagnosing cirrhosis and portal hypertension^3^. It detects liver morphology changes and specific signs of portal hypertension, including liver surface nodularity and portosystemic collaterals like paraumbilical veins and splenorenal collaterals, along with portal venous flow reversal^4,5^.

In grayscale ultrasound, the average normal portal vein diameter (PVD) is typically under 13 mm, with age and sex as considerations^6^. Portal vein velocity (PVV) has high sensitivity and a positive predictive value (PPV) for esophageal varices formation in cirrhosis. PVV with a rule-out cutoff value greater than 19 cm/s has 96% sensitivity for screening of esophageal varices^7^.

The splenic index, derived from three dimensions (anteroposterior, transverse, and craniocaudal) estimates splenic volume better than single measurements^8^. A normal range is 120 cm^3^ to 480 cm^3^, with values greater than 480 cm^3^ indicating splenomegaly^9^. The congestion index (CI) reflects the hemodynamic status of the portal venous system and is 2.5 times higher in cirrhotic subjects compared to normal individuals (average of 0.07±0.014 in normal and average of 0.104±0.039 in cirrhosis)^10^.

Esophageal endoscopy, the gold standard for upper gastrointestinal bleeding diagnosis and treatment, faces issues like invasiveness, complications, and rural accessibility. Noninvasive tools like abdominal ultrasound are preferred by patients, as they can predict esophageal varices in high-risk cirrhotic patients without invasive endoscopy^11–13^.

This study aims to provide evidence supporting the reliability and noninvasive nature of the ultrasonographic evaluation of portal vein and splenic indices to predict gastroesophageal varices in patients with liver cirrhosis.

## Materials and method

### Study design

This prospective, observational analytical study was carried out in the Department of Radiology and Imaging. The study was carried out from October 2020 to November 2021. Ethical approval for conducting the study was taken from the Institutional Review Committee (IRC) (approval number^6–11^ E^2^ 077/078. The work is reported in line with the Standard for Reporting of Diagnostic Accuracy Studies (STARD)^14^. The study is registered retrospectively in the research registry with a unique identification number (UIN) of researchregistry9180.

### Sample Size

A sample size of 64 was calculated using the sensitivity formula with a confidence level of 90%, a level of significance of 10%, the sensitivity of the test being 96%^7^ and a reasonable estimate of key proportions to be measured in the study was 16%^2^.

### Inclusion and exclusion criteria

Newly or previously diagnosed patients of cirrhosis the age of above 18 years based on their clinical, biochemical, histopathological, and ultrasound findings (increased hepatic parenchymal echogenicity, shrunken liver with irregular surface), presenting to the Department of Radiology, with a recent upper GI endoscopy report were included in the study.

Patients having cirrhosis with hepatic encephalopathy grade III and IV, noncirrhotic portal hypertension, extrahepatic portal vein obstruction, Budd Chiari syndrome, hepatocellular carcinoma, portal vein thrombosis, splenic hematological malignancy, receiving beta blocker, who had undergone splenectomy and those who did not provide consent were not included in the study.

### Data collection

**Figure 1:**
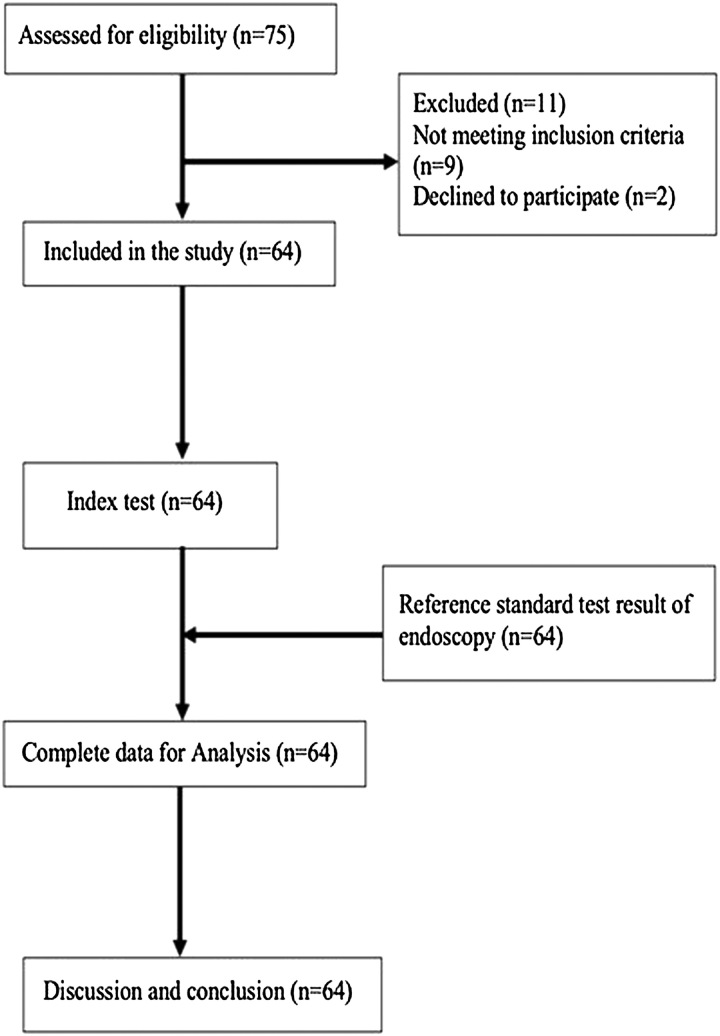
STARD flow diagram illustrating participant flow of the liver cirrhosis.

Stable participants referred from the Gastroenterology department to the Department of Radiology for liver cirrhosis evaluation were included who met the inclusion criteria (Fig. 1).

**Figure 2:**
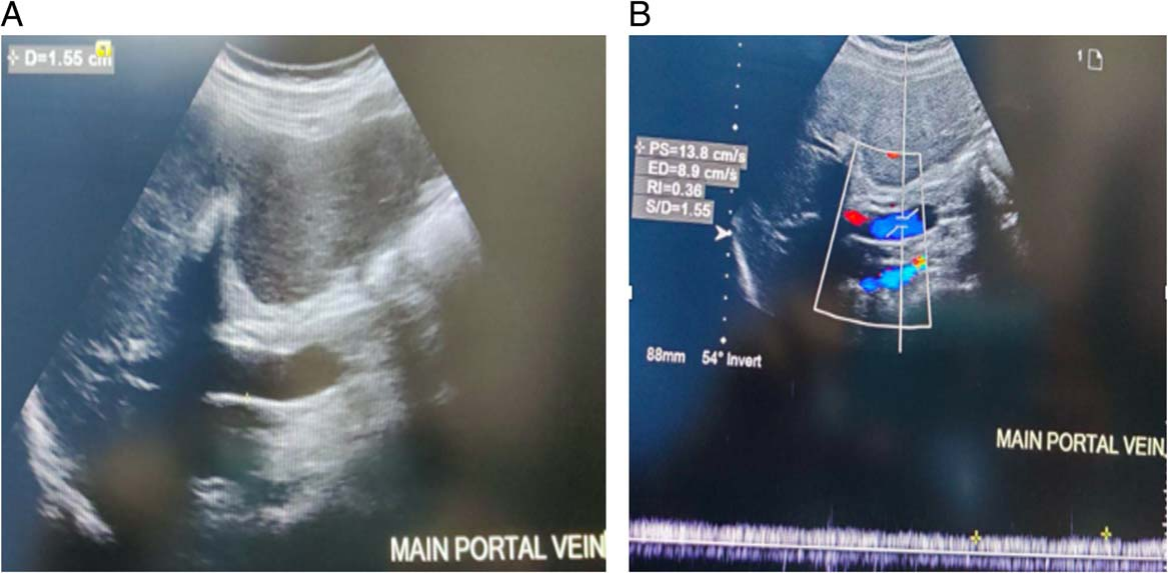
A: Greyscale ultrasound image showing the measurement of the portal vein diameter. B: Spectral Doppler ultrasound image showing maximum and minimum portal vein velocity measurement.

**Figure 3:**
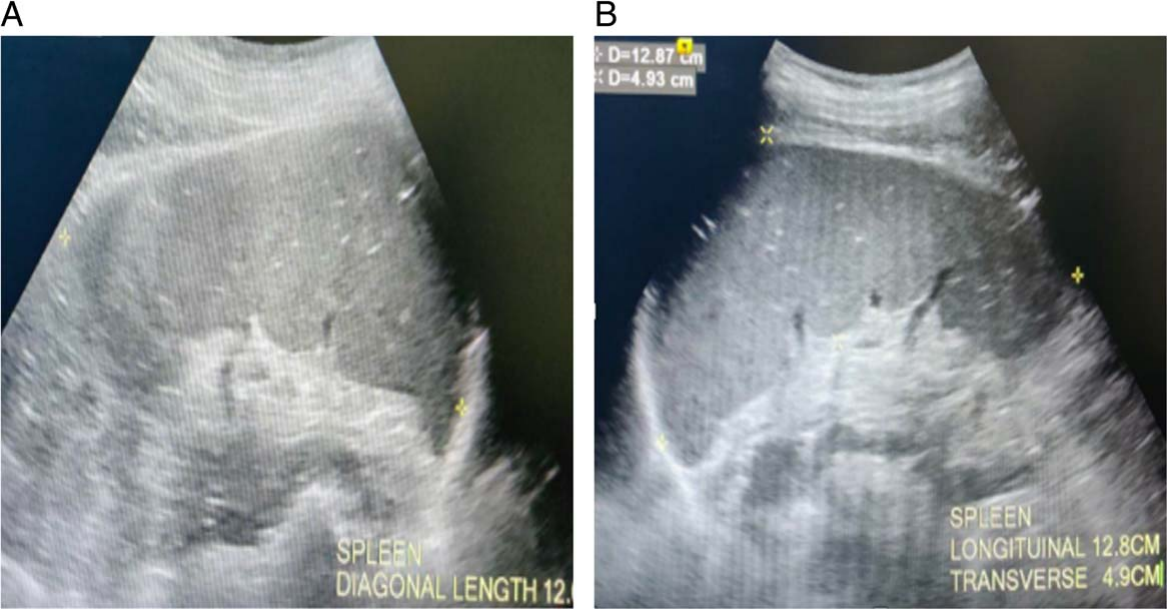
A: Greyscale ultrasound image showing the measurement of the diagonal dimension of the spleen. B: Greyscale ultrasound image showing the measurement of the longitudinal and transverse dimensions of the spleen.

A high-resolution real-time Doppler ultrasound scanner, equipped with a 3.5 MHz curvilinear transducer, was used to identify and measure the PVD and velocity using color Doppler (Fig. 2A and B). Experienced radiologists conducted the ultrasound examinations, and they were blinded to both the patients’ clinical histories and the reports from endoscopic examinations. The cross-sectional area and CI were calculated based on these measurements. Spleen size was measured in the supine position during full inspiration, longitudinal transverse, and diagonal dimensions were measured (Fig. 3A and B) and the splenic index was calculated by equation,

Splenic index=longitudinal diameter×transverse diameter×thickness×0.52

Endoscopy is performed in the Department of Gastroenterology to identify gastroesophageal varices and portal hypertension signs and the report was made available for the study to radiology department same day. Endoscopy offers direct visualization and accurate assessment of varices, which alternative imaging methods like CT may not offer. The American Association classifies esophageal varices into small and large sizes, with small sizes corresponding to Grades I and II and large sizes to Grades III and IV of the Paquet classification^15^.

A structured Performa was used to record the data prospectively. These techniques and measurements were employed to investigate the association between the portal vein and splenic indices and the presence of gastroesophageal varices in patients with liver cirrhosis.

### Statistical analysis

The study used IBM SPSS version 24 and Microsoft Excel software to analyze the data. Sensitivity, specificity, PPV, negative predictive value, and diagnostic accuracy were calculated for PVV, PVD, splenic index, CI with consideration of endoscopy as a gold standard test. A Pearson correlation test was performed between the quantitative indices to see their correlation. The correlation of the quantitative indices with variceal findings was tested after post-hoc correction using an unpaired *t*-test. The area under the receiver operating characteristic curve (AUROC) was studied to look for predictability.

## Result

This study included 64 subjects diagnosed with chronic liver disease and referred by a clinician for a routine follow-up scan consisting of male 38 (59%) and female 26 (41%). The most common etiology was alcoholic liver disease followed by viral hepatitis (Table 1).

**Table 1:** Clinico-demographic profile of patients with cirrhosis of the liver referred to the radiology department.

Characteristics	Number (%)
Sex	
Male	38 (59%)
Female	26 (41%)
Age	
>60 year	8 (13%)
50–59 year	18 (28%)
40–49 year	29 (45%)
30–39 year	9 (14%)
<30 year	0 (0%)
Clinical history	
Jaundice	27 (42.2%)
Abdominal distension	8 (12.5%)
Body swelling	28 (43.8%)
Pain abdomen	1 (1.5%)
Etiology of chronic liver disease	
Alcoholic liver disease	55 (86%)
Hepatitis B	5 (8%)
Hepatitis C	3 (4.5%)
Cryptogenic	1 (1.5%)
Endoscopic findings	
No varices	8 (12.5%)
Small varices	36 (56.25%)
Large varices	20 (31.25%)

The relationship between portal vein parameters, splenic index, and CI with varices was done, which showed the increased value of PVD, splenic index, and CI in patients with varices while PVV was higher in those without varices (Table 2).

**Table 2:** Measurements of the portal vein indices and splenic index parameter in respect to varices.

Measurement indices	Average value	Patient with gastroesophageal varices	Without gastroesophageal varices
Portal vein diameter	14.9±1.7 mm	14.7±1.64 mm	12.05±1.26 mm
Portal vein velocity	14.5±2.29 cm/s	13.91±2.01 cm/s	17.9±0.6 cm/s
Splenic index	1032±518 cm^3^	1120±494 cm^3^	419±89.3 cm^3^.
Congestion index	0.14±0.05 cm/s	0.15±0.05 cm/s	0.08±0.02 cm/s

PVV was found to have the highest sensitivity, followed by CI, splenic index, and PVD, with CI being the most accurate for assessing esophageal varices (Table 3).

**Table 3:** Diagnostic tests and their measurements.

Diagnostic tests (with endoscopy as gold standard)	Sensitivity	Specificity	PPV	NPV	LR+	LR-	Diagnostic accuracy
Splenic index (480 cm^3^ cutoff)	92.85%	87.5%	98.11%	63.63%	7.4	0.08	92.18%
Portal vein diameter (13 mm cutoff)	91.07	75%	96.2%	54.54%	3.64	0.11	89%
Portal vein velocity (19 cm/s as cutoff)	98.2%	37.5%	91.6%	75%	1.57	0.04	90.6%
Congestion index (0.10 as cutoff)	94%	87%	98%	70%	7.2	0.06	93.75%

**Figure 4:**
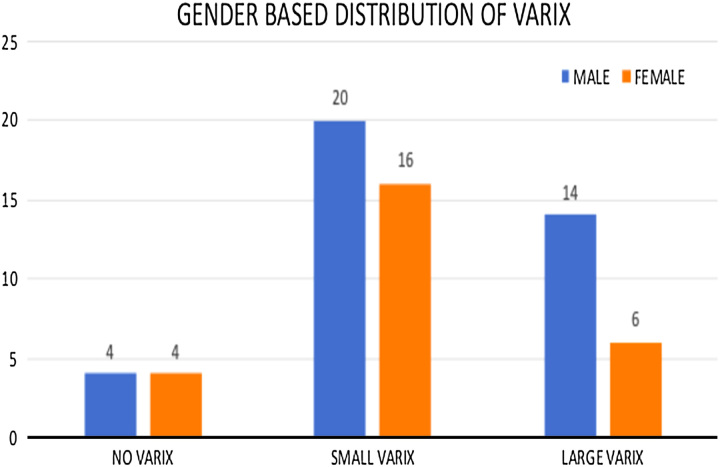
Histogram showing the sex-based distribution of the varix.

The study revealed that most individuals had small varices, with males having a higher incidence and larger varices more frequently observed (Fig. 4).

The study revealed significant correlations between PVD and splenic index, maximum PVV, and splenic index, and PVD and maximum PVV. Additionally, a significant correlation was found between the splenic index and the CI (Table 4).

**Table 4:** Pearson correlation of various portal vein indices and splenic index.

Pearson correlation of various Indices	Correlation coefficient (r value)	*P*
Portal vein diameter and splenic index	0.37	0.002
Portal vein velocity and splenic index	0.34	0.005
Portal vein diameter and portal vein velocity	0.36	0.003
Splenic index and congestion index	0.36	0.003

A one-way ANOVA test showed a significant correlation between the PVD and splenic index with various grades of esophageal varices with a *P*-value of <0.05. A significant correlation between PVV and CI with esophageal varices is also noted with a *P*-value of 0.0005 and 0.00004, respectively.

**Figure 5:**
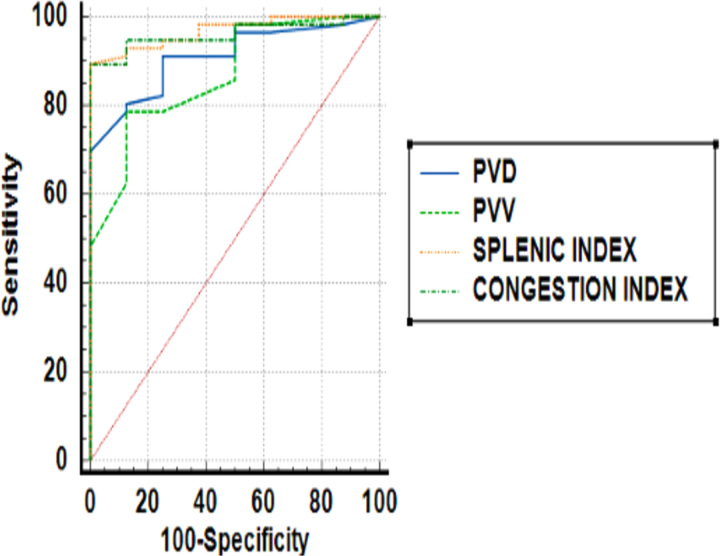
Receiver operating curve (ROC) analysis of the portal vein indices and splenic index.

Although all four indices were found to be the predictor of esophageal varices, the splenic index was found to be a better predictor with the highest AUROC of 96.8% followed by the CI, which accounts for about 96% (Fig. 5) (Table 5).

**Table 5:** Area under curve data for various indices.

Variable	Area under curve (AUC)	95% CI
PVD	0.91	0.81–0.96
PVV	0.86	0.76–0.94
Splenic index	0.968	0.89–0.99
Congestion index	0.960	0.88–0.99

These results suggest a potential sex difference in the presence and size of varices in liver cirrhosis patients.

## Discussion

Earlier screening of esophageal varices in cirrhotic patients with prophylactic and interventional treatment can reduce the incidence of variceal bleeding and reduce morbidity and mortality. Noninvasive radiological, clinical, or laboratory parameters can be used to predict the presence of esophageal varices^16^. This study was done to establish the role of the splenic index and portal vein indices in predicting gastroesophageal varices in cirrhotic patients.

PVD greater than 13 mm was considered dilatation irrespective of sex in this study. The study found 91.07% sensitivity and 96.2% PPV of PVD for the varices. A study done by Yazdi and Khalilian^17^ with the same cutoff value found 89% of sensitivity and 89% of specificity. The lower sensitivity compared to our study may be due to the small sample size of 36. Bhattarai *et al*.^18^ also found high sensitivity and specificity of PVD for predicting varices with greater than 12.25 mm rule-out cutoff value for the PVD. Shankar *et al*.^19^ kept the rule-out cutoff value of PVD of 12.2 mm and found a sensitivity of 80% and a specificity of 80% to predict the esophageal varices. Both were consistent with our findings. The low sensitivity in the study by Shankar *et al*. is probably due to a small sample size of 50 as compared to our study. This study compared PVV between two groups with and without varices confirmed endoscopically. Results showed lower PVV in subjects with varices than those without varices, with slightly lower PVV in males. The sensitivity, specificity, and PPV of PVV with a cutoff value of 19 cm/s were 98.2, 37.5, and 91.6%, respectively.

The study conducted by Elkenawy *et al*.^4^ with a cutoff value of 19 cm/s also showed high sensitivity of PVV for predicting esophageal varices with rule-out cutoff value of PVV less than 19 cm/s, with sensitivity being 97% and specificity of 40%. Shastri *et al*.^7^ concluded the fact that PVV can be used as the best index for the prediction of varices before sending the patient for endoscopy based on their study with the sensitivity of PVV being 84% with rule-out cutoff value of PVV taken as 16 cm/s. However, the sample size was smaller as compared to Elkenawy *et al.* (50 vs. 135). Our result showed the same sensitivity with a similar cutoff as Elkenawy *et al*.

The splenic index was recorded ultrasonographically and greater than 480 cm^3^ was considered enlarged size irrespective of sex. Subjects with varices were found to have higher splenic indexes compared to subjects without varices. The study showed a high AUROC of 96.8% and was statistically significantly similar to Saulat *et al*.^20^. The splenic index has high diagnostic accuracy, but the small sample size limits the specificity of our study. There is limited existing research concerning the evaluation of the sensitivity and specificity of the splenic index in relation to gastroesophageal varices.

CI greater than 0.10 cm/s was considered for portal hypertension, irrespective of sex, and was compared between two groups with and without varices confirmed endoscopically. It showed 94% of sensitivity, 87% specificity, 98% PPV and 93.75% of diagnostic accuracy. A study done by Moriyasu *et al*.^10^ with CI cutoff value similar to our study showed 93% sensitivity and 85.7% of specificity, which was very similar to our study. CI was also higher in subjects with varices compared to the nonvariceal group and more in male subjects. Statistically high significance was noted in the study of CI for the prediction of esophageal varices in a study performed by Hekmatnia *et al*.^21^ and Leao *et al*.^22^. They set the cutoff value at a slightly higher value compared to ours and the result was reduced specificity and diagnostic accuracy of the CI.

A significant correlation between the portal vein and splenic index seen in our study indicates that alteration in the portal vein characteristics can impact splenic and congestion indices providing insight into the hemodynamic changes associated with liver cirrhosis and varices.

## Limitation

The study was unable to grade the severity of liver damage due to biopsy findings. The portal vein was measured at the level where it crosses the inferior vena cava at the level 2 cm from the hilum, which does not address the anatomical variation. Single-center enrollment may affect the study generalization and intraobserver variability, but a highly trained physician with ultrasound being performed at the same time of day may reduce these limitations. The sample size used was small and only eight subjects had no varix. Noninvasive indices such as transient elastography can be added to better predict esophageal varices.

## Conclusion

Ultrasound is a noninvasive tool for diagnosing chronic liver disease and measuring portal vein indices (diameter and velocity) and splenic index. This study found increased PVD, CI, and splenic index but reduced average PVV in a patient with portal hypertension due to liver cirrhosis. Noninvasive indices like the splenic index and CI have higher diagnostic accuracy and AUROC, making them a good predictor for esophageal varices in cirrhosis of the liver. Early referral of the patient to the higher center could be done for upper gastrointestinal endoscopic management based on the measurement of these indices.

## Ethical approval

We have conducted an ethical approval base on the Declaration of Helsinki with registration research at the Institutional Review Committee (IRC) of the Institute of Medicine (IOM), Tribhuvan University, Nepal. Reference number: 76(6-11)E2 077/078.

## Consent

Written informed consent was obtained from the patient for the publication of this case report and the accompanying images. A copy of the written consent is available for review by the Editor-in-Chief of this journal on request.

## Sources of funding

None.

## Author contribution

S.K.: conceptualization, as mentor and reviewer for this original article and for data interpretation; M.A.A.: conceptualization and reviewer for this case; S.S.: reviewer and data interpretation; S.R. and P.G.: contributed in performing literature review and editing; A.G.: contributed in writing the paper and reviewer for this case. All authors have read and approved the manuscript.

## Conflicts of interest disclosures

All the authors declare that they have no competing interest.

## Research registration unique identifying number (UIN)

1. Name of the registry: researchregistry.com.
2. Unique identifying number or registration ID: researchregistry9180.
3. Hyperlink to your specific registration (must be publicly accessible and will be checked): https://www.researchregistry.com/register-now#home/registrationdetails/649488d0b325630028ade7fb/

## Guarantor

Shailendra Katwal is the person in charge of the publication of our manuscript.

## Data availability statement

Data sharing is not applicable to this article.

## Provenance and peer review

Not commissioned, externally peer-reviewed.
